# The Potential Benefit of 5% Sulfamylon Solution in the Treatment of *Acinetobacter baumannii*–contaminated Traumatic War Wounds

**Published:** 2005-02-22

**Authors:** John O. Kucan, John P. Heggers

**Affiliations:** a Southern Illinois University School of Medicine, Springfield; b The University of Texas Medical Branch and Shriners Hospitals for Children, Burns Hospital, Galveston

## Abstract

**Background:** The recent report of high numbers of *Acinetobacter baumannii* bloodstream infections among service members injured in Iraq and Afghanistan during the period January 2002 through August 2004 has prompted an investigation into their etiology. A review of the current guidelines for open combat casualty wounds as part of this broad investigation was not mentioned in the report. **Objective:** The objective of this study was 2-fold: to ascertain the susceptibility of *A baumannii* to currently available topical antibacterial agents and (2) to propose an alternative, effective treatment protocol for contaminated combat-related wounds so as to reduce or eliminate the likelihood of the wound serving as the source of *A baumannii* infection or septicemia. **Methods:** A standardized antimicrobial susceptibility study of 43 strains of *A baumannii* collected from a tertiary care burn center was conducted using 2 commonly used topical antibacterial agents, 1% silver sulfadiazine cream (Silvadene) and 5% mafenide acetate solution (5% Sulfamylon Solution). **Results:** Both were effective, but 5% Sulfamylon Solution demonstrated significantly greater antibacterial activity. **Conclusion:** Five percent Sulfamylon Solution, initially developed for wartime use, and currently limited by the Food and Drug Administration to soaks following meshed split-thickness autografts following excision of second-degree and third-degree burns, has a broad spectrum of antibacterial activity and extensive off-label applicability. It is an ideal agent for use in the treatment of war wounds, and should be considered as a superior replacement for normal saline in the current guidelines for open combat casualty wounds

The recent report of an increasing number of *Acinetobacter baumannii* bloodstream infections among service members injured in Iraq/Afghanistan operations prompted this investigation.^1^ The number of such infections and the resistance of *A baumannii* infections to multiple antibiotics suggested the need to consider and address the wounds and their treatment as a possible cause of septicemia and to suggest an alternative method to decrease or eliminate colonized or contaminated wounds as a potential source of *A baumannii* sepsis.

The present guidelines for care of open combat casualty wounds are as follows:

The standard care for the type of wounds we are seeing from Iraq include vigorous and complete early irrigation and debridement (Figs 1–3). The wounds are packed with saline-soaked Kling or fine mesh gauze and left alone for 4 days (Fig 4). The only reason to take down the dressing and inspect the wound is foul odor, discharge, bleeding, or fever that cannot be explained without inspecting the wound. At 4 days, the patient is returned to the operating room for a dressing change. If the wound has that “sticky” appearance without any areas of necrosis and need for further extensive debridement, a Delayed Primary Closure (DPC) is done. If the wound does not appear clean, it is debrided, irrigated, and packed again (sometimes with Dakin's solution or whatever solution the surgeon chooses) and the 4-day clock restarted (judgement call). Those wounds that fail DPC or are so large that a DPC is not in the plan are treated by wet-to-dry dressing changes. As soon as the wounds appear clean, Dakin's solution (or whatever solution is used to clean the wound) is switched *to normal saline*.^2^

Normal saline solution possesses no inherent antibacterial activity, and therefore cannot be expected to decrease or control bacterial growth in open wounds. Likewise, simple inspection of such wounds is unreliable in predicting successful DPC.^3^ Because systemic antibiotics are ineffective in reducing bacterial counts in granulating wounds, the use of a topical antibacterial agent that is active in controlling or reducing bacterial proliferation in open wounds may substantially decrease wound sepsis as a source of morbidity and may have a beneficial effect on overall management.^4^,^5^ Furthermore, the addition of microbiological quantification within the wounds (quantitative bacteriology or swab techniques) may provide valuable information regarding efficacy of treatment and the likelihood of successful DPC.^6^,^7^

The efficacy of 5% mafenide acetate solution (5% Sulfamylon Solution [Bertek Pharmaceutical Inc, Research Triangle Park, NC]) and 1% silver sulfadiazine cream (Silvadene [Kendall Company, Mansfield, Mass]) was tested against *A baumannii* derived from burn isolates at the Shriners' Burn Institute, Galveston, Tex, in order to determine whether these topical antibacterial agents, successfully employed in burn care, might be effective adjuncts in controlling *A baumannii* wound infections in traumatic war wounds.

## MATERIALS AND METHODS

All *A baumannii* isolates from the burn population were routinely tested for their susceptibility to 5% Sulfamylon Solution and Silvadene by a modified Nathan's agar well assay.

Mueller-Hinton 150 mm agar plates received 6 mm agar wells using a sterile 6 mm biopsy punch. Once the wells were made, 10^5^ colony-forming units of the organism were inoculated to each plate. The plates were allowed to dry, and then 0.1 mL of each topical antimicrobial was added to each well and numerically identified. A total of 43 *Acinetobacter* isolates were tested.

The assay plates were incubated at 37°C for 24 hours. Zones of inhibition were measured and compared for susceptibility.^8^

## RESULTS

Table 1 gives the average zones of inhibition for Silvadene and Sulfamylon against *A baumannii.* Sulfamylon treatment had a significantly greater zone of inhibition than did Silvadene treatment (*P* = .05).

### Statistical assessment

Zones of inhibition for the 2 topical antimicrobials were compared using a 1-way analysis of variance followed by a Tukey's test. Statistical significance was set at *P* < .05.

## DISCUSSION

The emergence of an increased number of bloodstream infections due to antibiotic-resistant *Abaumannii* among service members wounded during Operation Enduring Freedom (Afghanistan) and Operation Iraqi Freedom (Iraq/Kuwait) from the period January 2002 through August 2004 has focused attention on the issues of infection control in combat settings and healthcare facilities (field hospitals, combat theater medical facilities, military medical centers) and the need for new, effective antibiotics to treat these infections. Using the criteria established by the Centers for Disease Control and Prevention's National Nosocomial Infection Surveillance system, data compiled during this period identified 102 patients with blood cultures positive for *A baumannii.* Of the 102 service members, 85 (83%) sustained wounds during either military operation.^1^

*A baumannii* is ubiquitous and has recently become one of the most important healthcare-associated hospital pathogen, resulting in significant morbidity and mortality. Over the last 40 years, nosocomial infections caused by this agent have become increasingly problematic, posing significant therapeutic challenges due to multiple antibiotic resistance, persistent colonization, and prolonged environmental survivability. *A baumannii* has been the cause of infections in patients suffering from traumatic injuries and was the most common gram-negative bacillus isolated from traumatic extremity injuries during the Vietnam War.^9^–^14^ Therefore, environmental contamination of wounds with *A baumannii* must be considered as a possible source of infection. Presently, it is not known when or where military personnel who served in Operation Enduring Freedom or Operation Iraqi Freedom acquired the infections: in the field, during evacuation, or during treatment.

The information compiled on these patients is currently under review. Multiple factors, including antimicrobial exposure, mechanical ventilation, vascular access, and evacuation of patients through treatment facilities, are implicated. Microbiological and molecular analyses of patient and environmental isolates are being conducted to determine the likely sources of infection. However, neither the current standard guidelines for care of open combat casualty wounds have been reviewed nor has any consideration been given to assess more effective ways to ensure bacteriologic control of the wounds to reduce the possibility of the wound serving as the septic source.^1^

The present guidelines for the care of open combat casualty wounds require vigorous irrigation, debridement, saline-soaked dressings, and reinspection at 96 hours, unless otherwise required. DPC is performed on wounds with that sticky appearance. Wounds not amenable to DPC undergo additional wound care and dressing changes employing wet-to-dry dressings, Dakin's solution or “whatever solution the surgeon chooses,” and normal saline.^2^

The purpose of this study was 2-fold: (1) to ascertain the susceptibility of *A baumannii* to currently available topical antibacterial agents and (2) to propose an alternative, effective treatment protocol for contaminated combat-related wounds so as to reduce or eliminate the likelihood of the wound serving as the source of *A baumannii* infection or septicemia.

This study showed the efficacy of 2 commonly used topical antibacterial agents, 1% silver sulfadiazine cream (Silvadene) and 5% mafenide acetate solution (5% Sulfamylon Solution), against a large number of strains of *A baumannii.* The latter demonstrated significantly greater activity against *A baumannii* than did 1% silver sulfadiazine cream.

Topical antibacterial agents are routinely employed in burn care to prevent burn wound sepsis, but their use in nonburn wounds has been generally limited and underappreciated. Nevertheless, these agents, and 5% mafenide acetate solution in particular, have been effectively utilized in the management of nonburn wounds and should be considered as important adjuncts in the management of acute and chronic open wounds. The product indications for 5% Sulfamylon Solution have been severely limited by the Food and Drug Administration to use over meshed split-thickness skin grafts following excision of second-degree and third-degree burn wounds. However, the overall utility of this agent in off-label indications, including contaminated and infected wounds, has been clearly demonstrated since World War II and is unsurpassed.^15^–^17^

The demonstration in this study of the antibacterial efficacy of 5% mafenide acetate solution against *A baumannii* is neither an accident nor serendipity. Mafenide, either as hydrochloride or as acetate, has been shown to be an effective topical antibacterial agent since its adoption by the German Army in World War II. Numerous studies conducted by Dr Janice Mendelson and by Drs John Moncrief and Robert Lindberg at the US Army Institute of Surgical Research, Brooke Army Medical Center, Fort Sam Houston, Tex, confirmed its effectiveness in the research laboratory and in the clinical setting.^17^–^21^

Five percent mafenide acetate solution (5% Sulfamylon Solution) is especially effective in the treatment of burns, blast injuries, open fractures, synergistic gangrene, and necrotizing fasciitis. It retains its activity in the presence of blood and pus, and can penetrate beneath the surface of the wound to exert its antibacterial effects on both viable and nonviable tissue. It is not cytotoxic, may stimulate angiogenesis, and exerts its activity directly at the wound site. It is highly effective against both gram-positive and gram-negative bacteria, including anaerobes. It has no antifungal activity. Studies of the agent conducted against numerous strains of gram-positive and gram-negative organisms, including methicillin-resistant *Staphylococcus aureus*, vancomycin-resistant enterococcus, *Pseudomonas aeruginosa*, and *Acinetobacer* species, isolated from a broad array of clinical situations showed complete activity. In another study of more than 11,000 strains of *P aeruginosa*, collected over 25 years at the US Army Institute of Surgical Research, Brooke Army Medical Center, Fort Sam Houston, Tex, there was no evidence of resistance to 5% Sulfamylon Solution.^15^–^28^

Mafenide acetate powder is stable and autoclavable and requires no refrigeration. It is supplied in drums and is then dispensed in 50 g premeasured packets of powder. Fifty grams of powder is mixed with 1 L of sterile water to yield a 5% solution of the agent. The resultant solution is clear and colorless and can be applied to wounds in the same fashion as is normal saline. Rather then relying on a single application of normal saline, which has no antibacterial activity and may predispose the wound to bacterial proliferation, 5% Sulfamylon Solution exerts a powerful, continuous antibacterial effect on a broad range of bacteria, including *A baumannii*. The dressings need to be remoistened with Sulfamylon Solution at regular intervals (6–8 hours) to prevent wound dessication and to maintain antibacterial potency.^15^,^27^

This agent was developed for wartime requirements and is the ideal topical agent for wound care in difficult environments. It is highly effective against the pathogens most commonly encountered in massive traumatic injuries, blasts, gunshot wounds, burns, and open fractures. Because of its aqueous nature it can be easily incorporated into dressings as a soak and it permits easy inspection of the wound. It can be applied with spray bottles or injected into rubber catheters that have been incorporated into the dressings in order to maintain antibacterial control.

The inclusion of bacteriological monitoring of the wound (quantitative bacteriology or swab techniques) into the overall wound management protocol may be helpful in gauging the efficacy of wound treatment and the suitability of the wound for DPC.^3^,^7^

## CONCLUSION

The increased incidence of *A baumannii* septicemia involving service members wounded during Operation Iraqi Freedom and Operation Enduring Freedom has prompted a thorough investigation to determine the source of the infections and to decrease their likelihood in the future. The investigation is focusing on many variables and factors that may play a causative role. Current treatment guidelines for open combat casualty wounds were not mentioned as part of the review.

The standard protocol currently in use relies on the fundamental concepts of adequate debridement and copious wound irrigation but does not provide for ongoing antibacterial activity within the wound prior to DPC.

Five percent Sulfamylon Solution was tested against numerous *A baumannii* isolates and exerted a strong antibacterial effect as demonstrated by large zones of inhibition in a standardized microbial susceptibility assay. The historical and current clinical and laboratory data clearly support the efficacy and utility of this topical agent in a wide variety of soft tissue wounds and infections. This agent was initially developed for wartime use and should be considered as part of the battlefield casualty antibacterial armamentarium. By substituting this agent for normal saline in the treatment protocol of open combat casualty wounds, reliable and effective bacteriologic control of the wound from the entire spectrum of battlefield bacterial contaminants (gram-positive and gram-negative) can be achieved.

## Figures and Tables

**Figure 1:**
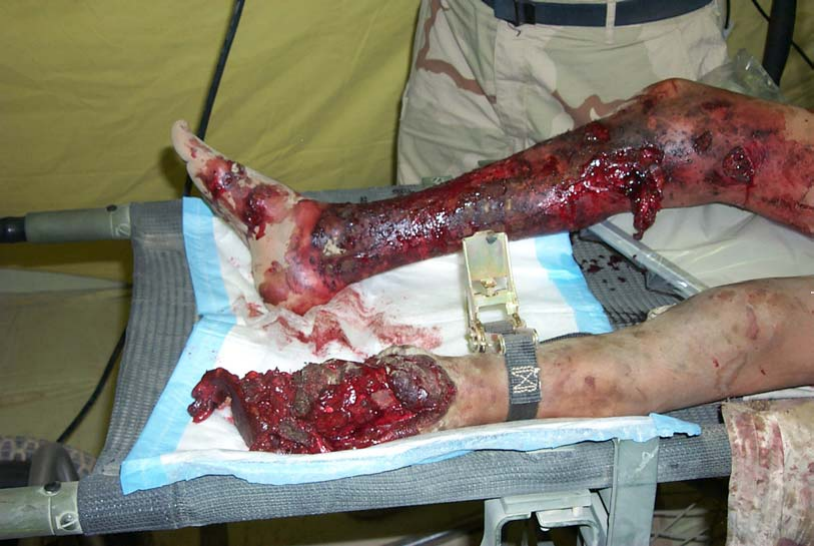
Patient with burns, traumatic amputation left foot and lower leg, and multiple soft tissue injuries secondary to blast (Afghanistan, 2003).

**Figure 2:**
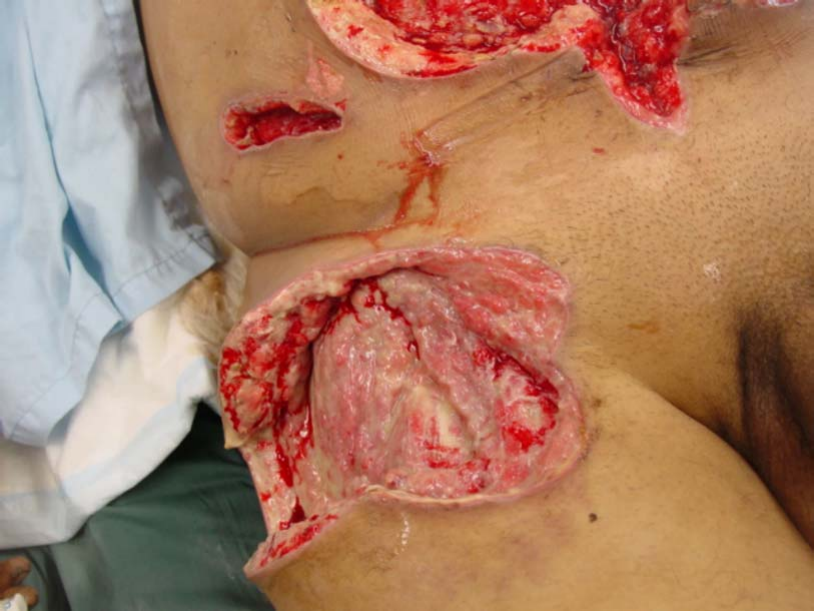
Debrided shrapnel wounds to right thigh, flank, and abdomen (Afghanistan, 2003).

**Figure 3:**
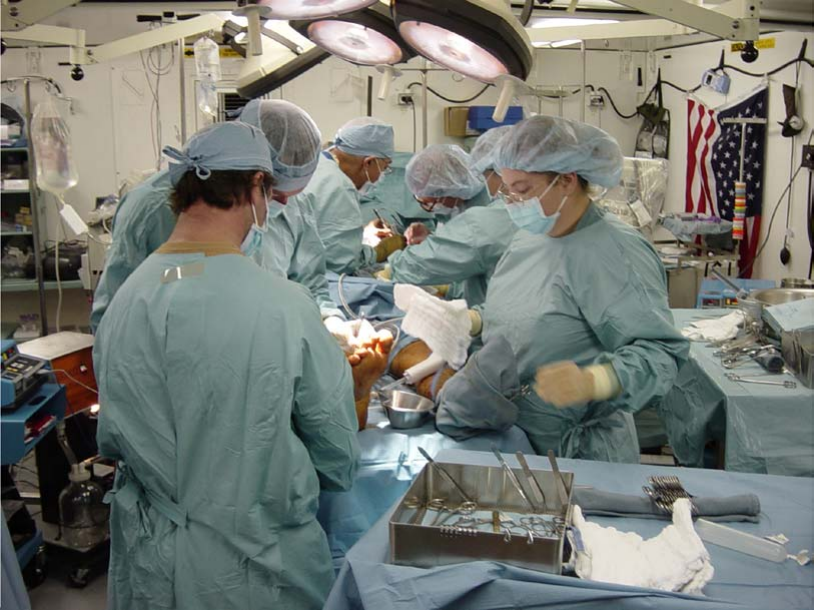
Surgical team in action (Afghanistan, 2003).

**Figure 4:**
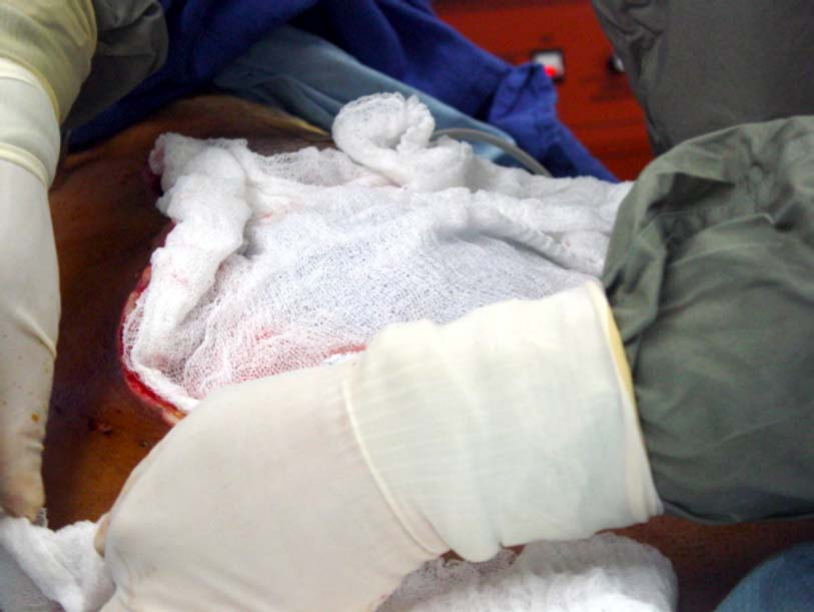
Normal saline–soaked dressing being applied to open abdominal wound (Afghanistan, 2003).

**Table 1:** Average ± standard error of inhibition for Silvadene and Sulfamylon against Acinetobacter baumannii

		**Average ± standard error of inhibition, mm**		
		
**Organism**	**Total number**	**Silvadene**	**Sulfamylon**	**Value**
*A baumannii*	43	15 ± 1	23 ± 1	*P* < .05
